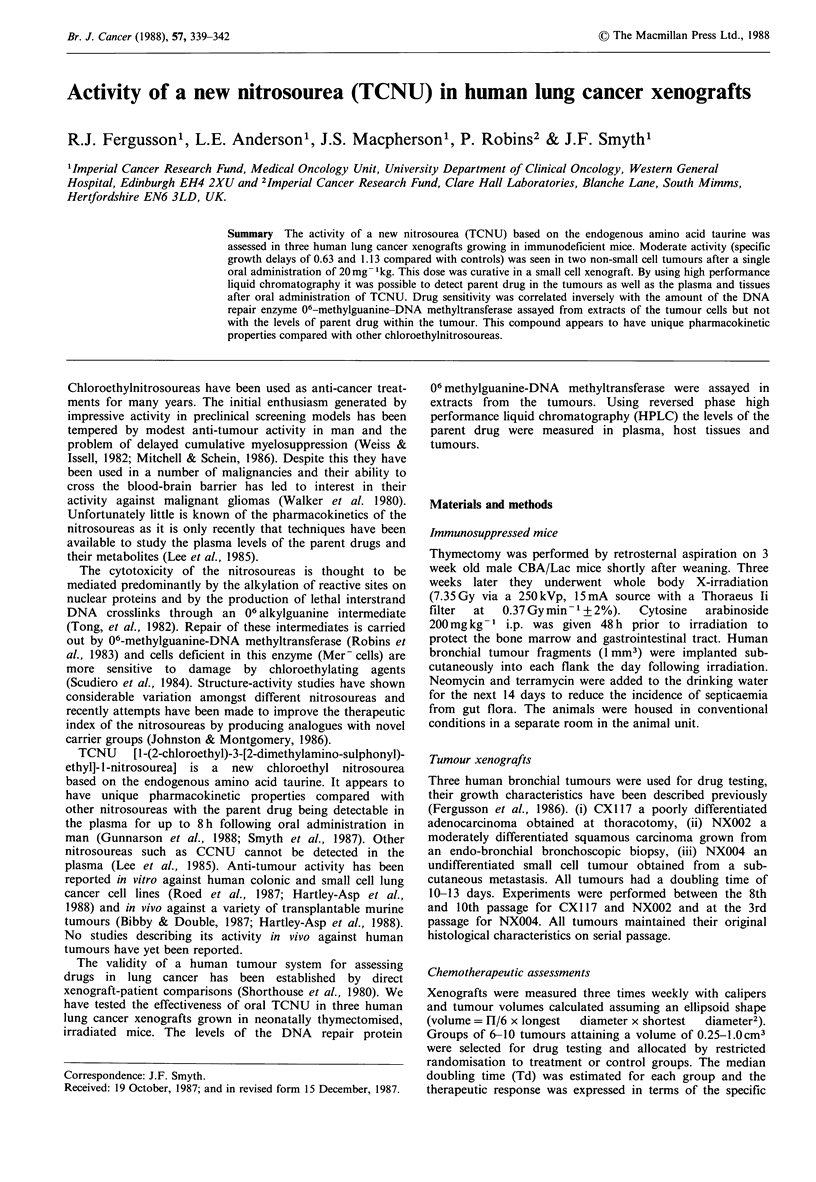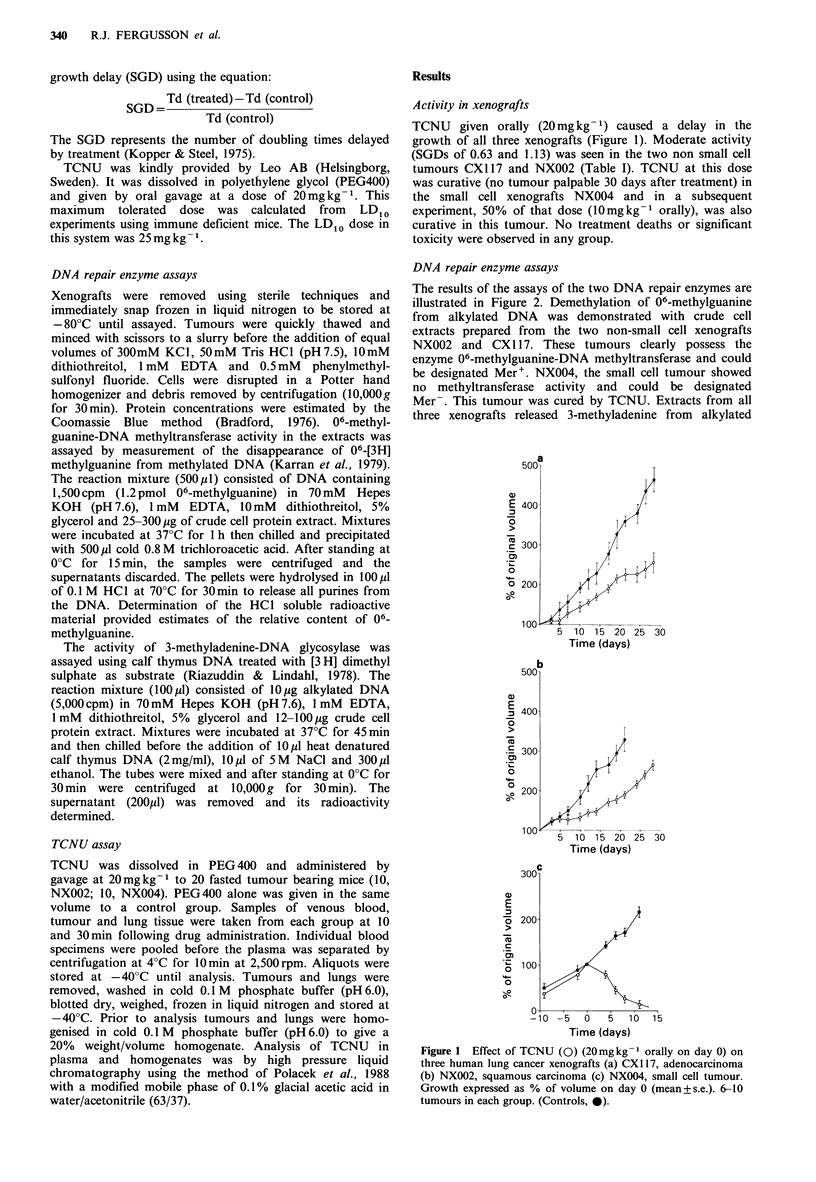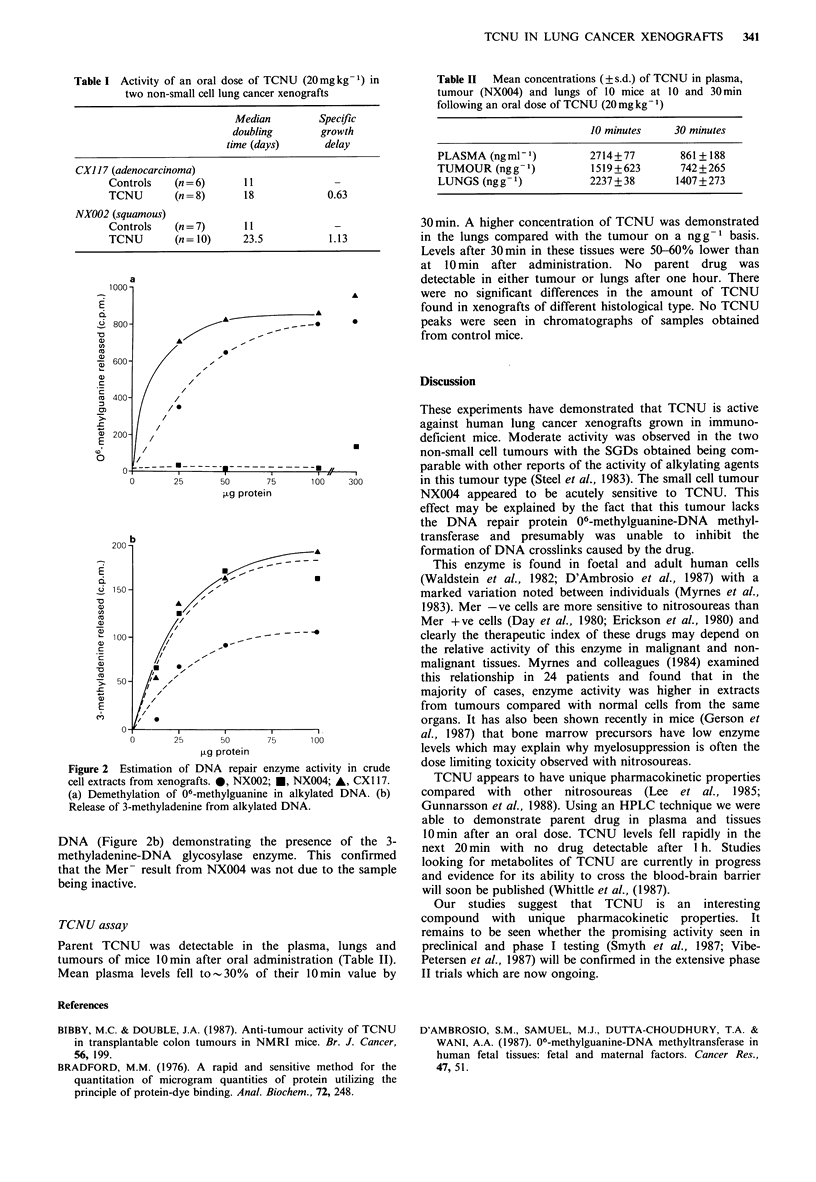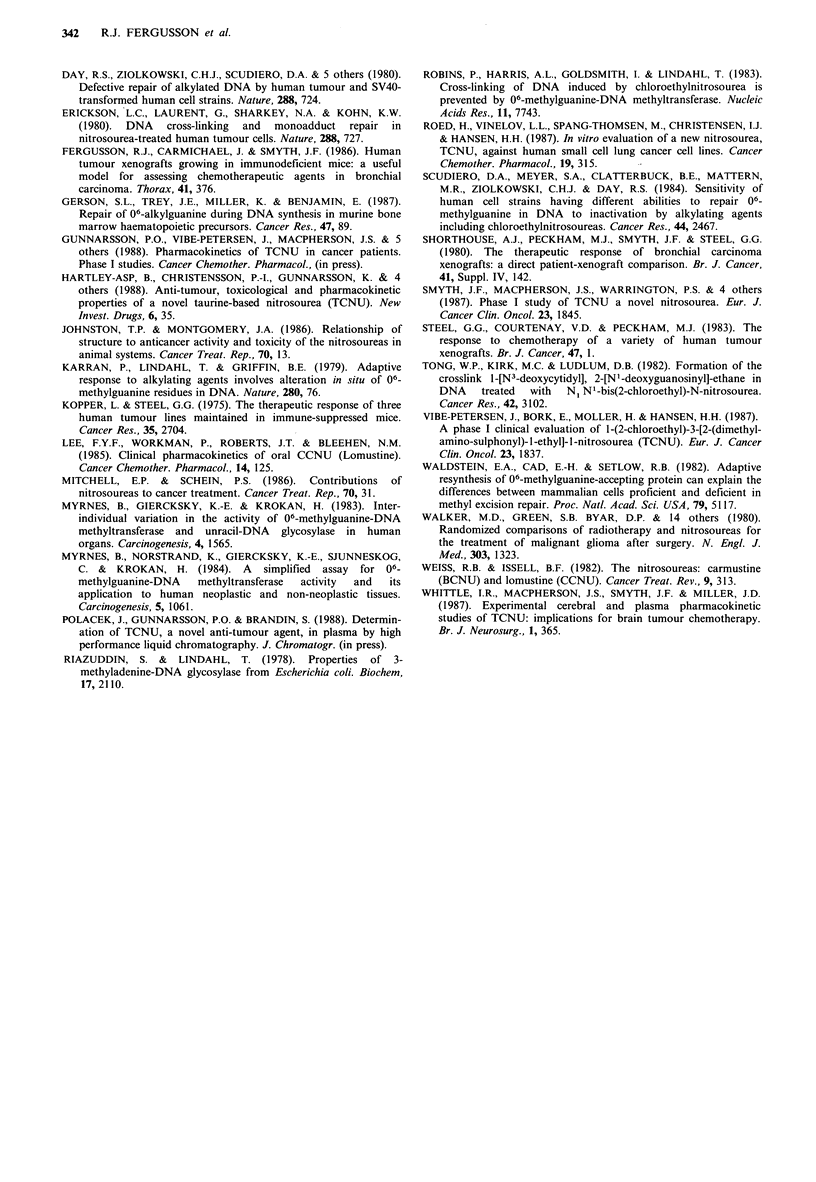# Activity of a new nitrosourea (TCNU) in human lung cancer xenografts.

**DOI:** 10.1038/bjc.1988.78

**Published:** 1988-04

**Authors:** R. J. Fergusson, L. E. Anderson, J. S. Macpherson, P. Robins, J. F. Smyth

**Affiliations:** University Department of Clinical Oncology, Western General Hospital, Edinburgh, UK.

## Abstract

The activity of a new nitrosourea (TCNU) based on the endogenous amino acid taurine was assessed in three human lung cancer xenografts growing in immunodeficient mice. Moderate activity (specific growth delays of 0.63 and 1.13 compared with controls) was seen in two non-small cell tumours after a single oral administration of 20 mg-1kg. This dose was curative in a small cell xenograft. By using high performance liquid chromatography it was possible to detect parent drug in the tumours as well as the plasma and tissues after oral administration of TCNU. Drug sensitivity was correlated inversely with the amount of the DNA repair enzyme 0(6)-methylguanine-DNA methyltransferase assayed from extracts of the tumour cells but not with the levels of parent drug within the tumour. This compound appears to have unique pharmacokinetic properties compared with other chloroethylnitrosoureas.


Br. J. Cancer (1988), 57, 339–342 © The Macmillan Press Ltd., 1988

### Activity of a new nitrosourea (TCNU) in human lung cancer xenografts

R.J. Fergusson[1], L.E. Anderson[1], J.S. Macpherson[1], P. Robins[2] & J.F. Smyth[1]

[1]*Imperial Cancer Research Fund, Medical Oncology Unit, University Department of Clinical Oncology, Western General Hospital, Edinburgh EH4 2XU and* [2]*Imperial Cancer Research Fund, Clare Hall Laboratories, Blanche Lane, South Mimms, Hertfordshire EN6 3LD, UK.*

**Summary** The activity of a new nitrosourea (TCNU) based on the endogenous amino acid taurine was assessed in three human lung cancer xenografts growing in immunodeficient mice. Moderate activity (specific growth delays of 0.63 and 1.13 compared with controls) was seen in two non-small cell tumours after a single oral administration of $20\,mg^{-1}kg$. This dose was curative in a small cell xenograft. By using high performance liquid chromatography it was possible to detect parent drug in the tumours as well as the plasma and tissues after oral administration of TCNU. Drug sensitivity was correlated inversely with the amount of the DNA repair enzyme $0^6$–methylguanine–DNA methyltransferase assayed from extracts of the tumour cells but not with the levels of parent drug within the tumour. This compound appears to have unique pharmacokinetic properties compared with other chloroethylnitrosoureas.

Chloroethylnitrosoureas have been used as anti-cancer treatments for many years. The initial enthusiasm generated by impressive activity in preclinical screening models has been tempered by modest anti-tumour activity in man and the problem of delayed cumulative myelosuppression (Weiss & Issell, 1982; Mitchell & Schein, 1986). Despite this they have been used in a number of malignancies and their ability to cross the blood-brain barrier has led to interest in their activity against malignant gliomas (Walker *et al.* 1980). Unfortunately little is known of the pharmacokinetics of the nitrosoureas as it is only recently that techniques have been available to study the plasma levels of the parent drugs and their metabolites (Lee *et al.*, 1985).

The cytotoxicity of the nitrosoureas is thought to be mediated predominantly by the alkylation of reactive sites on nuclear proteins and by the production of lethal interstrand DNA crosslinks through an $0^6$ alkylguanine intermediate (Tong, *et al.*, 1982). Repair of these intermediates is carried out by $0^6$-methylguanine-DNA methyltransferase (Robins *et al.*, 1983) and cells deficient in this enzyme ($Mer^-$ cells) are more sensitive to damage by chloroethylating agents (Scudiero *et al.*, 1984). Structure-activity studies have shown considerable variation amongst different nitrosoureas and recently attempts have been made to improve the therapeutic index of the nitrosoureas by producing analogues with novel carrier groups (Johnston & Montgomery, 1986).

TCNU [1-(2-chloroethyl)-3-[2-dimethylamino-sulphonyl)-ethyl]-1-nitrosourea] is a new chloroethyl nitrosourea based on the endogenous amino acid taurine. It appears to have unique pharmacokinetic properties compared with other nitrosoureas with the parent drug being detectable in the plasma for up to 8 h following oral administration in man (Gunnarson *et al.*, 1988; Smyth *et al.*, 1987). Other nitrosoureas such as CCNU cannot be detected in the plasma (Lee *et al.*, 1985). Anti-tumour activity has been reported *in vitro* against human colonic and small cell lung cancer cell lines (Roed *et al.*, 1987; Hartley-Asp *et al.*, 1988) and *in vivo* against a variety of transplantable murine tumours (Bibby & Double, 1987; Hartley-Asp *et al.*, 1988). No studies describing its activity *in vivo* against human tumours have yet been reported.

The validity of a human tumour system for assessing drugs in lung cancer has been established by direct xenograft-patient comparisons (Shorthouse *et al.*, 1980). We have tested the effectiveness of oral TCNU in three human lung cancer xenografts grown in neonatally thymectomised, irradiated mice. The levels of the DNA repair protein $0^6$ methylguanine-DNA methyltransferase were assayed in extracts from the tumours. Using reversed phase high performance liquid chromatography (HPLC) the levels of the parent drug were measured in plasma, host tissues and tumours.

Correspondence: J.F. Smyth.
Received: 19 October, 1987; and in revised form 15 December, 1987.

### Materials and methods

### *Immunosuppressed mice*

Thymectomy was performed by retrosternal aspiration on 3 week old male CBA/Lac mice shortly after weaning. Three weeks later they underwent whole body X-irradiation (7.35 Gy via a 250 kVp, 15 mA source with a Thoraeus Ii filter at $0.37\,Gy\,min^{-1} \pm 2\%$). Cytosine arabinoside $200\,mg\,kg^{-1}$ i.p. was given 48 h prior to irradiation to protect the bone marrow and gastrointestinal tract. Human bronchial tumour fragments ($1\,mm^3$) were implanted subcutaneously into each flank the day following irradiation. Neomycin and terramycin were added to the drinking water for the next 14 days to reduce the incidence of septicaemia from gut flora. The animals were housed in conventional conditions in a separate room in the animal unit.

### *Tumour xenografts*

Three human bronchial tumours were used for drug testing, their growth characteristics have been described previously (Fergusson *et al.*, 1986). (i) CX117 a poorly differentiated adenocarcinoma obtained at thoracotomy, (ii) NX002 a moderately differentiated squamous carcinoma grown from an endo-bronchial bronchoscopic biopsy, (iii) NX004 an undifferentiated small cell tumour obtained from a subcutaneous metastasis. All tumours had a doubling time of 10–13 days. Experiments were performed between the 8th and 10th passage for CX117 and NX002 and at the 3rd passage for NX004. All tumours maintained their original histological characteristics on serial passage.

### *Chemotherapeutic assessments*

Xenografts were measured three times weekly with calipers and tumour volumes calculated assuming an ellipsoid shape (volume $= \Pi/6 \times$ longest diameter $\times$ shortest diameter$^2$). Groups of 6–10 tumours attaining a volume of $0.25–1.0\,cm^3$ were selected for drug testing and allocated by restricted randomisation to treatment or control groups. The median doubling time (Td) was estimated for each group and the therapeutic response was expressed in terms of the specific growth delay (SGD) using the equation:

$$SGD = \frac{\text{Td (treated)} - \text{Td (control)}}{\text{Td (control)}}$$

The SGD represents the number of doubling times delayed by treatment (Kopper & Steel, 1975).

TCNU was kindly provided by Leo AB (Helsingborg, Sweden). It was dissolved in polyethylene glycol (PEG400) and given by oral gavage at a dose of $20\,mg\,kg^{-1}$. This maximum tolerated dose was calculated from $LD_{10}$ experiments using immune deficient mice. The $LD_{10}$ dose in this system was $25\,mg\,kg^{-1}$.

*DNA repair enzyme assays*

Xenografts were removed using sterile techniques and immediately snap frozen in liquid nitrogen to be stored at −80°C until assayed. Tumours were quickly thawed and minced with scissors to a slurry before the addition of equal volumes of 300mM KCl, 50mM Tris HCl (pH7.5), 10mM dithiothreitol, 1mM EDTA and 0.5mM phenylmethylsulfonyl fluoride. Cells were disrupted in a Potter hand homogenizer and debris removed by centrifugation (10,000*g* for 30min). Protein concentrations were estimated by the Coomassie Blue method (Bradford, 1976). $0^6$-methylguanine-DNA methyltransferase activity in the extracts was assayed by measurement of the disappearance of $0^6$-[3H] methylguanine from methylated DNA (Karran *et al.*, 1979). The reaction mixture (500μl) consisted of DNA containing 1,500cpm (1.2pmol $0^6$-methylguanine) in 70mM Hepes KOH (pH7.6), 1mM EDTA, 10mM dithiothreitol, 5% glycerol and 25–300μg of crude cell protein extract. Mixtures were incubated at 37°C for 1h then chilled and precipitated with 500μl cold 0.8M trichloroacetic acid. After standing at 0°C for 15min, the samples were centrifuged and the supernatants discarded. The pellets were hydrolysed in 100μl of 0.1M HCl at 70°C for 30min to release all purines from the DNA. Determination of the HCl soluble radioactive material provided estimates of the relative content of $0^6$-methylguanine.

The activity of 3-methyladenine-DNA glycosylase was assayed using calf thymus DNA treated with [3H] dimethyl sulphate as substrate (Riazuddin & Lindahl, 1978). The reaction mixture (100μl) consisted of 10μg alkylated DNA (5,000cpm) in 70mM Hepes KOH (pH7.6), 1mM EDTA, 1mM dithiothreitol, 5% glycerol and 12–100μg crude cell protein extract. Mixtures were incubated at 37°C for 45min and then chilled before the addition of 10μl heat denatured calf thymus DNA (2mg/ml), 10μl of 5M NaCl and 300μl ethanol. The tubes were mixed and after standing at 0°C for 30min were centrifuged at 10,000*g* for 30min). The supernatant (200μl) was removed and its radioactivity determined.

*TCNU assay*

TCNU was dissolved in PEG400 and administered by gavage at $20\,mg\,kg^{-1}$ to 20 fasted tumour bearing mice (10, NX002; 10, NX004). PEG400 alone was given in the same volume to a control group. Samples of venous blood, tumour and lung tissue were taken from each group at 10 and 30min following drug administration. Individual blood specimens were pooled before the plasma was separated by centrifugation at 4°C for 10min at 2,500rpm. Aliquots were stored at −40°C until analysis. Tumours and lungs were removed, washed in cold 0.1M phosphate buffer (pH6.0), blotted dry, weighed, frozen in liquid nitrogen and stored at −40°C. Prior to analysis tumours and lungs were homogenised in cold 0.1M phosphate buffer (pH6.0) to give a 20% weight/volume homogenate. Analysis of TCNU in plasma and homogenates was by high pressure liquid chromatography using the method of Polacek *et al.*, 1988 with a modified mobile phase of 0.1% glacial acetic acid in water/acetonitrile (63/37).

### Results

*Activity in xenografts*

TCNU given orally ($20\,mg\,kg^{-1}$) caused a delay in the growth of all three xenografts (Figure 1). Moderate activity (SGDs of 0.63 and 1.13) was seen in the two non small cell tumours CX117 and NX002 (Table I). TCNU at this dose was curative (no tumour palpable 30 days after treatment) in the small cell xenografts NX004 and in a subsequent experiment, 50% of that dose ($10\,mg\,kg^{-1}$ orally), was also curative in this tumour. No treatment deaths or significant toxicity were observed in any group.

*DNA repair enzyme assays*

The results of the assays of the two DNA repair enzymes are illustrated in Figure 2. Demethylation of $0^6$-methylguanine from alkylated DNA was demonstrated with crude cell extracts prepared from the two non-small cell xenografts NX002 and CX117. These tumours clearly possess the enzyme $0^6$-methylguanine-DNA methyltransferase and could be designated $Mer^+$. NX004, the small cell tumour showed no methyltransferase activity and could be designated $Mer^-$. This tumour was cured by TCNU. Extracts from all three xenografts released 3-methyladenine from alkylated

**Figure 1** Effect of TCNU (○) ($20\,mg\,kg^{-1}$ orally on day 0) on three human lung cancer xenografts (a) CX117, adenocarcinoma (b) NX002, squamous carcinoma (c) NX004, small cell tumour. Growth expressed as % of volume on day 0 (mean±s.e.). 6–10 tumours in each group. (Controls, ●).

**Table I** Activity of an oral dose of TCNU (20 mg kg$^{-1}$) in two non-small cell lung cancer xenografts

| | | *Median doubling time (days)* | *Specific growth delay* |
|---|---|---|---|
| *CX117 (adenocarcinoma)* | | | |
| Controls | ($n$=6) | 11 | – |
| TCNU | ($n$=8) | 18 | 0.63 |
| *NX002 (squamous)* | | | |
| Controls | ($n$=7) | 11 | – |
| TCNU | ($n$=10) | 23.5 | 1.13 |

**Figure 2** Estimation of DNA repair enzyme activity in crude cell extracts from xenografts. ●, NX002; ■, NX004; ▲, CX117. (a) Demethylation of $0^6$-methylguanine in alkylated DNA. (b) Release of 3-methyladenine from alkylated DNA.

DNA (Figure 2b) demonstrating the presence of the 3-methyladenine-DNA glycosylase enzyme. This confirmed that the Mer$^-$ result from NX004 was not due to the sample being inactive.

### *TCNU assay*

Parent TCNU was detectable in the plasma, lungs and tumours of mice 10 min after oral administration (Table II). Mean plasma levels fell to ~30% of their 10 min value by

**Table II** Mean concentrations (±s.d.) of TCNU in plasma, tumour (NX004) and lungs of 10 mice at 10 and 30 min following an oral dose of TCNU (20 mg kg$^{-1}$)

| | *10 minutes* | *30 minutes* |
|---|---|---|
| PLASMA (ng ml$^{-1}$) | 2714±77 | 861±188 |
| TUMOUR (ng g$^{-1}$) | 1519±623 | 742±265 |
| LUNGS (ng g$^{-1}$) | 2237±38 | 1407±273 |

30 min. A higher concentration of TCNU was demonstrated in the lungs compared with the tumour on a ng g$^{-1}$ basis. Levels after 30 min in these tissues were 50–60% lower than at 10 min after administration. No parent drug was detectable in either tumour or lungs after one hour. There were no significant differences in the amount of TCNU found in xenografts of different histological type. No TCNU peaks were seen in chromatographs of samples obtained from control mice.

### Discussion

These experiments have demonstrated that TCNU is active against human lung cancer xenografts grown in immuno-deficient mice. Moderate activity was observed in the two non-small cell tumours with the SGDs obtained being comparable with other reports of the activity of alkylating agents in this tumour type (Steel *et al.*, 1983). The small cell tumour NX004 appeared to be acutely sensitive to TCNU. This effect may be explained by the fact that this tumour lacks the DNA repair protein $0^6$-methylguanine-DNA methyl-transferase and presumably was unable to inhibit the formation of DNA crosslinks caused by the drug.

This enzyme is found in foetal and adult human cells (Waldstein *et al.*, 1982; D'Ambrosio *et al.*, 1987) with a marked variation noted between individuals (Myrnes *et al.*, 1983). Mer −ve cells are more sensitive to nitrosoureas than Mer +ve cells (Day *et al.*, 1980; Erickson *et al.*, 1980) and clearly the therapeutic index of these drugs may depend on the relative activity of this enzyme in malignant and non-malignant tissues. Myrnes and colleagues (1984) examined this relationship in 24 patients and found that in the majority of cases, enzyme activity was higher in extracts from tumours compared with normal cells from the same organs. It has also been shown recently in mice (Gerson *et al.*, 1987) that bone marrow precursors have low enzyme levels which may explain why myelosuppression is often the dose limiting toxicity observed with nitrosoureas.

TCNU appears to have unique pharmacokinetic properties compared with other nitrosoureas (Lee *et al.*, 1985; Gunnarsson *et al.*, 1988). Using an HPLC technique we were able to demonstrate parent drug in plasma and tissues 10 min after an oral dose. TCNU levels fell rapidly in the next 20 min with no drug detectable after 1 h. Studies looking for metabolites of TCNU are currently in progress and evidence for its ability to cross the blood-brain barrier will soon be published (Whittle *et al.*, (1987).

Our studies suggest that TCNU is an interesting compound with unique pharmacokinetic properties. It remains to be seen whether the promising activity seen in preclinical and phase I testing (Smyth *et al.*, 1987; Vibe-Petersen *et al.*, 1987) will be confirmed in the extensive phase II trials which are now ongoing.

### References

BIBBY, M.C. & DOUBLE, J.A. (1987). Anti-tumour activity of TCNU in transplantable colon tumours in NMRI mice. *Br. J. Cancer*, **56**, 199.

BRADFORD, M.M. (1976). A rapid and sensitive method for the quantitation of microgram quantities of protein utilizing the principle of protein-dye binding. *Anal. Biochem.*, **72**, 248.

D'AMBROSIO, S.M., SAMUEL, M.J., DUTTA-CHOUDHURY, T.A. & WANI, A.A. (1987). $0^6$-methylguanine-DNA methyltransferase in human fetal tissues: fetal and maternal factors. *Cancer Res.*, **47**, 51.

DAY, R.S., ZIOLKOWSKI, C.H.J., SCUDIERO, D.A. & 5 others (1980). Defective repair of alkylated DNA by human tumour and SV40-transformed human cell strains. *Nature*, **288**, 724.

ERICKSON, L.C., LAURENT, G., SHARKEY, N.A. & KOHN, K.W. (1980). DNA cross-linking and monoadduct repair in nitrosourea-treated human tumour cells. *Nature*, **288**, 727.

FERGUSSON, R.J., CARMICHAEL, J. & SMYTH, J.F. (1986). Human tumour xenografts growing in immunodeficient mice: a useful model for assessing chemotherapeutic agents in bronchial carcinoma. *Thorax*, **41**, 376.

GERSON, S.L., TREY, J.E., MILLER, K. & BENJAMIN, E. (1987). Repair of $0^6$-alkylguanine during DNA synthesis in murine bone marrow haematopoietic precursors. *Cancer Res.*, **47**, 89.

GUNNARSSON, P.O., VIBE-PETERSEN, J., MACPHERSON, J.S. & 5 others (1988). Pharmacokinetics of TCNU in cancer patients. Phase I studies. *Cancer Chemother. Pharmacol.*, (in press).

HARTLEY-ASP, B., CHRISTENSSON, P.-I., GUNNARSSON, K. & 4 others (1988). Anti-tumour, toxicological and pharmacokinetic properties of a novel taurine-based nitrosourea (TCNU). *New Invest. Drugs*, **6**, 35.

JOHNSTON, T.P. & MONTGOMERY, J.A. (1986). Relationship of structure to anticancer activity and toxicity of the nitrosoureas in animal systems. *Cancer Treat. Rep.*, **70**, 13.

KARRAN, P., LINDAHL, T. & GRIFFIN, B.E. (1979). Adaptive response to alkylating agents involves alteration *in situ* of $0^6$-methylguanine residues in DNA. *Nature*, **280**, 76.

KOPPER, L. & STEEL, G.G. (1975). The therapeutic response of three human tumour lines maintained in immune-suppressed mice. *Cancer Res.*, **35**, 2704.

LEE, F.Y.F., WORKMAN, P., ROBERTS, J.T. & BLEEHEN, N.M. (1985). Clinical pharmacokinetics of oral CCNU (Lomustine). *Cancer Chemother. Pharmacol.*, **14**, 125.

MITCHELL, E.P. & SCHEIN, P.S. (1986). Contributions of nitrosoureas to cancer treatment. *Cancer Treat. Rep.*, **70**, 31.

MYRNES, B., GIERCKSKY, K.-E. & KROKAN, H. (1983). Inter-individual variation in the activity of $0^6$-methylguanine-DNA methyltransferase and unracil-DNA glycosylase in human organs. *Carcinogenesis*, **4**, 1565.

MYRNES, B., NORSTRAND, K., GIERCKSKY, K.-E., SJUNNESKOG, C. & KROKAN, H. (1984). A simplified assay for $0^6$-methylguanine-DNA methyltransferase activity and its application to human neoplastic and non-neoplastic tissues. *Carcinogenesis*, **5**, 1061.

POLACEK, J., GUNNARSSON, P.O. & BRANDIN, S. (1988). Determination of TCNU, a novel anti-tumour agent, in plasma by high performance liquid chromatography. *J. Chromatogr.* (in press).

RIAZUDDIN, S. & LINDAHL, T. (1978). Properties of 3-methyladenine-DNA glycosylase from *Escherichia coli*. *Biochem*, **17**, 2110.

ROBINS, P., HARRIS, A.L., GOLDSMITH, I. & LINDAHL, T. (1983). Cross-linking of DNA induced by chloroethylnitrosourea is prevented by $0^6$-methylguanine-DNA methyltransferase. *Nucleic Acids Res.*, **11**, 7743.

ROED, H., VINELOV, L.L., SPANG-THOMSEN, M., CHRISTENSEN, I.J. & HANSEN, H.H. (1987). *In vitro* evaluation of a new nitrosourea, TCNU, against human small cell lung cancer cell lines. *Cancer Chemother. Pharmacol.*, **19**, 315.

SCUDIERO, D.A., MEYER, S.A., CLATTERBUCK, B.E., MATTERN, M.R., ZIOLKOWSKI, C.H.J. & DAY, R.S. (1984). Sensitivity of human cell strains having different abilities to repair $0^6$-methylguanine in DNA to inactivation by alkylating agents including chloroethylnitrosoureas. *Cancer Res.*, **44**, 2467.

SHORTHOUSE, A.J., PECKHAM, M.J., SMYTH, J.F. & STEEL, G.G. (1980). The therapeutic response of bronchial carcinoma xenografts: a direct patient-xenograft comparison. *Br. J. Cancer*, **41**, Suppl. IV, 142.

SMYTH, J.F., MACPHERSON, J.S., WARRINGTON, P.S. & 4 others (1987). Phase I study of TCNU a novel nitrosourea. *Eur. J. Cancer Clin. Oncol.* **23**, 1845.

STEEL, G.G., COURTENAY, V.D. & PECKHAM, M.J. (1983). The response to chemotherapy of a variety of human tumour xenografts. *Br. J. Cancer*, **47**, 1.

TONG, W.P., KIRK, M.C. & LUDLUM, D.B. (1982). Formation of the crosslink 1-[$N^3$-deoxycytidyl], 2-[$N^1$-deoxyguanosinyl]-ethane in DNA treated with $N_1 N^1$-bis(2-chloroethyl)-N-nitrosourea. *Cancer Res.*, **42**, 3102.

VIBE-PETERSEN, J., BORK, E., MOLLER, H. & HANSEN, H.H. (1987). A phase I clinical evaluation of 1-(2-chloroethyl)-3-[2-(dimethyl-amino-sulphonyl)-1-ethyl]-1-nitrosourea (TCNU). *Eur. J. Cancer Clin. Oncol.* **23**, 1837.

WALDSTEIN, E.A., CAD, E.-H. & SETLOW, R.B. (1982). Adaptive resynthesis of $0^6$-methylguanine-accepting protein can explain the differences between mammalian cells proficient and deficient in methyl excision repair. *Proc. Natl. Acad. Sci. USA*, **79**, 5117.

WALKER, M.D., GREEN, S.B. BYAR, D.P. & 14 others (1980). Randomized comparisons of radiotherapy and nitrosoureas for the treatment of malignant glioma after surgery. *N. Engl. J. Med.*, **303**, 1323.

WEISS, R.B. & ISSELL, B.F. (1982). The nitrosoureas: carmustine (BCNU) and lomustine (CCNU). *Cancer Treat. Rev.*, **9**, 313.

WHITTLE, I.R., MACPHERSON, J.S., SMYTH, J.F. & MILLER, J.D. (1987). Experimental cerebral and plasma pharmacokinetic studies of TCNU: implications for brain tumour chemotherapy. *Br. J. Neurosurg.*, **1**, 365.